# Modes of Transportation to Medical and Primary Care Among Older Adults

**DOI:** 10.1093/geroni/igab046.490

**Published:** 2021-12-17

**Authors:** Zainab Suntai, Kefentse Kubanga, Emmanuel Adanu, Abhay Lidbe

**Affiliations:** University of Alabama, Tuscaloosa, Alabama, United States

## Abstract

Transportation is an increasingly meaningful concern for older adults as physical, cognitive, and psychological changes in older adulthood impact mobility and accessibility. While several studies have examined the modes of transportation used among older adults, few have explored specifically how older adults are accessing primary care/medical care services. As such, this study aimed to determine the specific modes of transportation used among older adults for primary care visits. Data were derived from the 2018 National Health and Aging Trends Study (NHATS), an annual longitudinal panel survey of older adults aged 65 and older living in the United States. Descriptive analyses were conducted to examine the prevalence of several modes of access and logistic regression models were used to predict the likelihood of using the two most prevalent transportation modes, based on sociodemographic and socioeconomic factors. Results showed that 70% of older adults drive themselves to their doctor, 34.8% rely on a family member, friend, or paid person, 2.4% have a home visit, 2.1% use public transportation, 1.5% walk to their doctor and 1.1% use a taxi. Additionally, having higher income, being of younger age, being White, and having post-secondary education was associated with driving oneself to the doctor. These results indicate that while most older adults are still self-reliant on transportation to medical providers, those with lower socioeconomic status are particularly at risk of losing driving independence. Transportation-related interventions should therefore consider targeting individuals with lower economic capital by proving financial assistance, ride-share programs, and other innovative approaches.

